# Impact of the CareWell integrated care model for older patients with multimorbidity: a quasi-experimental controlled study in the Basque *Country*

**DOI:** 10.1186/s12913-020-05473-2

**Published:** 2020-07-03

**Authors:** Maider Mateo-Abad, Nerea González, Ane Fullaondo, Marisa Merino, Lierni Azkargorta, Anna Giné, Dolores Verdoy, Itziar Vergara, Esteban de Manuel Keenoy

**Affiliations:** 1 Kronikgune Institute for Health Services Research, Barakaldo, Basque Country Spain; 2 Health Services Research on Chronic Patients Network (REDISSEC), Barakaldo, Basque Country Spain; 3 Osakidetza Basque Health Service, Hospital Galdakao-Usansolo, Galdakao, Basque Country Spain; 4 Osakidetza Basque Health Service, Tolosaldea Integrated Health Care Organization, Tolosa, Basque Country Spain; 5 Biodonostia Health Research Institute, Economic Evaluation of Chronic Diseases Group, Donostia, Basque Country Spain; 6 Biodonostia Health Research Institute, Primary Care Group, Donostia, Basque Country Spain

**Keywords:** Integrated care, Older, Multimorbidity, Care coordination, Implementation, Mixed-method, ICT, Patient empowerment, Home support

## Abstract

**Background:**

Older patients with multimorbidity have complex health and social care needs, associated with elevated use of health care resources. The aim of this study is to evaluate the impact of CareWell integrated care model for older patients with multimorbidity in the Basque Country.

**Methods:**

The CareWell program for older patients with multimorbidity, based on the coordination between health providers, home-based care and patient empowerment, supported by information and communication technology tools. The program was deployed in four healthcare areas in the Basque Country. The control group was formed by two organizations in which the program had not been deployed and regular care procedures were applied.

Participants, older patients (aged ≥65) with two or more chronic conditions (at least one being chronic obstructive pulmonary disease, chronic heart failure, or diabetes mellitus), categorized as complex according to a risk stratification algorithm, were followed up to 12 months. The impact of the program on the use of health resources, clinical effectiveness, and satisfaction was evaluated using a mixed-method approach.

Semi-structured interviews were performed to assess satisfaction with the newly deployed model and mixed regression models to measure the effect of the intervention throughout the follow-up period.

**Results:**

Two hundred patients were recruited (101 intervention and 99 control), mostly males (63%) with a mean age of 79 years and age-adjusted Charlson Comorbidity Index of 9.7 on average. Relevant differences between the groups were observed for all dimensions. In the intervention group, the number of hospitalizations and visits to emergency centers was reduced, and the number of primary care contacts increased. Clinical changes were also observed, such as a decrease in the body mass index and blood glucose levels. The satisfaction level was high for all stakeholders.

**Conclusion:**

The implementation of CareWell integrated care model changed the profile of health resource utilization, strengthening the key role of primary care and reducing the number of emergency visits and hospitalizations. The satisfaction with this model of care was high.

**Trial registration:**

ClinicalTrials.gov, NCT03042039. Registered 3 February 2017 - Retrospectively registered.

## Background

A growing proportion of the population in OECD countries is aged 65 and over: 15% in 2010 and expected to reach 22% by 2030. In 2010, in the Basque Country, more than 19% of the population was older than 65 (currently, over 21%) [1]. More than the 45% of the Basque population has at least one chronic condition; this percentage increases with age, exceeding 80% in people over 65 years of age [2]. Older multimorbid patients have complex health and social care needs, are at risk of hospital or residential care-home admission, and require many high-level interventions [3]. Aging and chronic conditions are associated with 80% of the medical consultations in the Basque health system, accounting for 77% of the total health budget [4].

The Basque healthcare system (Osakidetza) is run using a National Health Services (NHS) model, often dubbed the Beveridge type. This public healthcare model is funded by general taxation and offers medical cover to all residents, with a target population of 3 million. Despite this apparently integrated (in management terms) system, at the provider level, the actual integration of services has not achieved yet the expected integrated clinical care and care continuity [4]. The system is hindered by fragmentation, lack of coordination between healthcare levels and inability to provide the continuity of care required for good management of complex patients living with multiple chronic conditions. Around 25% of the population perceive primary and specialized care as uncoordinated [2].

To meet these challenges, in 2010, a new strategy to tackle chronicity was proposed by the Department of Health of the Government of the Basque Country [5]. The need to implement new organizational models was noted. Since then, the Basque health system, Osakidetza, has deployed a specific strategy to improve the structural integration and care coordination [4], considered and promoted by the World Health Organization during recent years [6]. These changes have been applied progressively in some of the organizations within the overall health system. Integrated care approaches are more effective and efficient in ensuring the quality and continuity of care [7], however, the suboptimal implementation processes, leads to their diminished efficacy. There is some evidence of combining different strategies into a broad program of care [8].

This project, within the framework of the European CareWell project [9], proposes to design, implement and assess the deployment of an integrated care program based on patient empowerment, home-support pathway and coordination between health providers. It is supported by a wide range of information and communication technology (ICT) tools. This program has been developed and deployed in six European regions.

The care coordination pathway was designed to facilitate communication, role coordination and sequencing of the activities of the multidisciplinary care team, using the ICT tools as enablers [10]. Optimal collaboration and coordination between professionals in the delivery of integrated care have become essential for the provision of high-quality care [11]. Care-transition support is considered a priority in optimal care [12]. Difficulties in transition can lead not only to the deterioration in patient care but also to ‘bed-blocking’ and lack of efficiency [13].

The ICT platforms and communication channels allow proper alignment, avoid duplication of effort and bridge gaps in patient care. Interoperable ICT systems improve the process by making the professionals aware of patient care in its holistic sense. It can also augment surveillance and physician performance measures [14].

The aim of this study was to evaluate, in the Basque Country, the impact of the CareWell integrated care model for older patients with multimorbidity, using quantitative and qualitative techniques.

## Methods

### Study design

This is a quasi-experimental study using intervention and control groups. The intervention group was drawn from patients registered in four healthcare organizations, where the new integrated care pathway was deployed. The control group consisted of patients registered in two organizations providing the usual care. A mixed-method approach has been used to evaluate the effect of the program in terms of use of health resources, clinical effectiveness, patient functional and mental status and satisfaction. This mixed-method technique integrates and analyzes the data employing both, quantitative and qualitative methods, to examine the same research issues, through different methodological perspectives and from the point of view of the main stakeholders [15].

### Participants

A group of 200 older complex patients (≥ 65 years old), with two or more chronic conditions (with at least one of them a chronic obstructive pulmonary disease (COPD), chronic heart failure (CHF) or diabetes mellitus) was recruited. Complexity of patients was an inclusion criterion, defined as having a predictive risk index higher than 6.28, according to the Basque population-based risk stratification. This meant that, for these patients, the probability of using the health services in the following year was at least 6.28 times higher than for an average Basque citizen [16].

Exclusion criteria were a severe mental or physical condition preventing the use of questionnaires, terminal illness, or lack of consent to participate in the study. The patients in the intervention group came from four integrated care organizations (ICO), Tolosaldea ICO, Ezkerraldea-Enkarterri-Cruces ICO, Bilbao-Basurto ICO, and Uribe ICO. The patients in the control group were recruited in Barrualde-Galdakao ICO and Araba ICO. The patients were identified, approached and invited to participate in the study by their General Practitioner (GP) or the Primary Care (PC) Nurse. All the included participants were able to understand and comply with study instructions, either independently or with help from their carer. All participants provided their written informed consent.

For the qualitative evaluation, a random sample was selected from each group of stakeholders involved in the main study. This included the patients, their informal carers, health professionals (physicians and nurses) responsible for their care, and leaders of the participating health centers. Whenever the randomization was not possible, a convenience sampling strategy was employed, inviting the stakeholders who might have more specific information on the issues to be explored.

### Intervention

#### Usual care

Patients in the control group received the usual care. Primary Care professionals, GPs and PC nurses, are responsible for most of the healthcare activities performed at the community and home levels, such as on demand consultation, home visits, drug prescription, patient education, or referral to the specialist or hospital care. The primary care is based on well-equipped facilities with 100% coverage of electronic health records (EHR) and e-prescription. A 24 × 7 eHealth call center, staffed by trained nurses, is available to respond to phone calls from patients. They follow validated protocols, and can access the EHRs of the patients. If admitted to the hospital, a patient with multimorbidity can be allocated to one of the specialist wards. A dedicated consultant can coordinate other specialists during the hospitalization period. Discharge is coordinated between the hospital liaison nurse and the PC nurse.

#### CareWell integrated care pathway

**Fig. 1:**
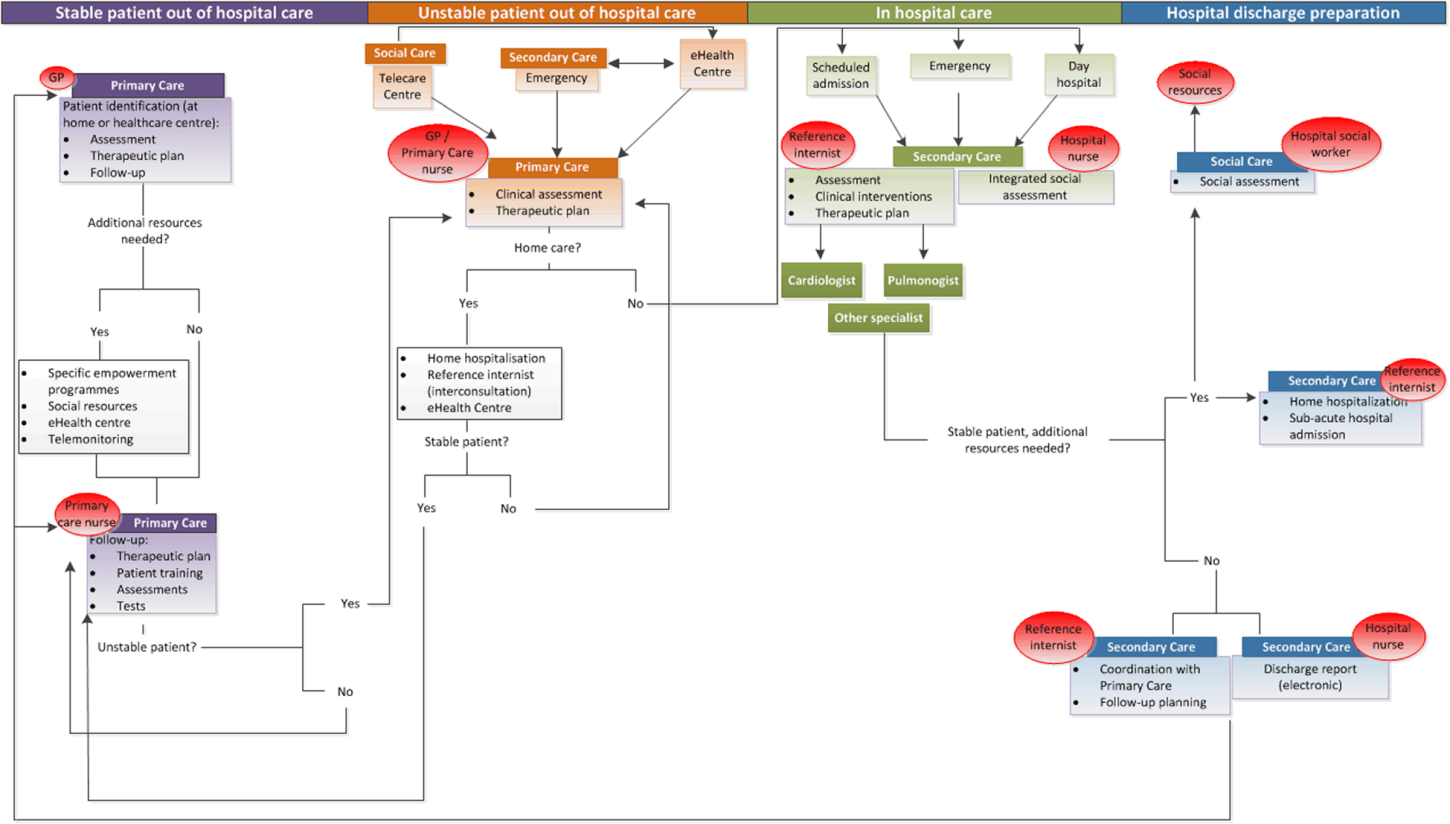
CareWell program pathway in the Basque Country. Diagram of the newly deployed integrated organizational model. Patients were stratified into different levels of interventions using agreed criteria for care intensification or specific actions to perform under particular circumstances

The CareWell integrated care model has defined a specific pathway for patients with multimorbidity, in addition to the usual care. It has several phases: identification of frail older patients, comprehensive baseline assessment, definition of the therapeutic plan, programmed follow-up, patient stabilization at home, integrated care during hospitalization and coordinated hospital discharge. The pathway focuses on two main dimensions: 1) care coordination and communication between health providers and 2) patient empowerment and home-based care. These are supported by ICT-based platforms, including a Personal Health Folder, which allows the patients to access their clinical information [17]. A diagram defining the integrated organizational model followed in the Basque Country is shown in Fig. 1.

The multidisciplinary team, usually in charge of the patient, deployed clear roles based on patient status, sharing explicit decision-making information. This information includes scales and indicators used in the clinical assessment, referrals, follow-up and social-need detection or identification of reinforcements necessary for patient empowerment. The multidisciplinary teams include the following profiles: the General Practitioner, the Social Worker, the Specialists, the Nurse Care Manager, and the eHealth Center. The Nurse Care Manager is responsible not only for the specific case management but also supports the patients in the hospital, emergency department, and during the discharge process. The roles of the Reference Internist and the Hospital Liaison Nurse were reinforced. A personalized plan drawn for each patient, includes follow-up within 24–48 h after discharge and monthly telephone calls by the PC nurse to allow early detection of possible deterioration. Messaging between patients and/or carers and healthcare practitioners via the Personal Health Folder was enabled.

A patient empowerment program, KronikOn [18], was defined. The KronikOn targets frail older patients and their carers. It includes a basic set of four 20- to 30-min sessions at the health center or at home. Primary and secondary care nurses provide essential information to help the patients to understand their condition and to explore and agree upon the best methods of self-care. The patients have web access to KronikOn educational material.

### Data collection

The baseline data collection took place between May and December 2015, and the participants were followed-up for a period of 9 to 12 months. Some sociodemographic and clinical data were collected during (not blinded) personal interviews performed by a trained nurse, at the baseline and at the end of the follow-up period. The EHR and administrative databases were then used to extract automatically other available information on clinical outcomes (at the two time points), and on the use of services (throughout the follow-up period).

The qualitative evaluation took place after the follow-up period to obtain in-depth information on the pros and cons of the new integrated model, the main barriers and facilitators in its implementation. Semi-structured interviews were used to compare the evaluation results between the main stakeholders, they were developed ad-hoc for this study (Additional file 1). Nine interviews were performed: 2 with patients, 2 with carers, 2 with clinicians, 2 with nurses and 1 with a manager. The mean length of an interview was 38 min; they were conducted by two people, one asking the questions and the other taking notes on the conduct of the meeting. The interviews were recorded, with the consent of the participant, and transcribed afterward.

### Variables

Sociodemographic and lifestyle characteristics were gender, age, marital status, level of education, smoking habits, and use of devices, mobile phones and personal computers.

The use of health services was studied by examining the number of contacts with health care providers (GPs, nurses, specialists and others), with the hospital (including duration of hospitalizations) and visits to emergency centers.

Clinical variables included the diagnosed chronic conditions (included in the Charlson Comorbidity Index [19]) and health-related parameters such as body mass index (BMI), blood pressure (mmHg), heart rate (bpm), oxygen saturation (%), blood glucose (mg/dl), HbA1c (%), and creatinine (mg/dl); the last two parameters were used only when appropriate. Mental status was examined using the short form of the Geriatric Depression Scale (GDS) [20], in which a score of 5 or more points indicates depression symptoms. Functional status was assessed using the Barthel Index [21, 22], which ranges from 0 to 100, where lower scores indicate increased dependency.

For the user perspective, the interviews were designed to address the following variables: care plan, care coordination, and management of the disease employing the ICTs.

### Statistical analysis

Categorical variables are presented using the frequencies and percentages, n (%). Differences between groups are analyzed employing the χ^2^ test. Continuous variables with a normal distribution are presented as means and standard deviations (SD), and the differences between groups are examined using the Student’s *t*-test. Non-normal distributed continuous variables are presented as median and the first and third quartile (Q1, Q3), and the differences are examined using nonparametric Wilcoxon rank-sum test. Pre–post differences for categorical variables are calculated using McNemar’s test for paired data. For continuous variables, Student’s *t*-test and Wilcoxon signed-rank test for paired data are used for normal and non-normal distributed variables, respectively.

Generalized regression models were used to analyze the CareWell effect for different outcomes. These models are adjusted by the specific outcome baseline value, age, gender, follow-up period, degree of comorbidity measured by Charlson Comorbidity Index, and the BMI baseline value. Linear multivariate regression was performed for continuous outcomes, and multivariate logistic regression, for discrete outcomes. The results show the difference between intervention over control group, with its corresponding confidence interval of 95% and the *p*-value. All the analyses were performed using the free-software R v. 3.4.0.

The core information for the qualitative analyses was the text of each interview. This text was the fusion of the notes taken during the interviews and the literal transcriptions made from the recordings of each meeting.

A thematic analysis of narratives was made using the inductive method of reading and re-coding, to generate an explanatory framework obtained from the empirical data. The thematic analysis has been described as “a method for identifying, analyzing and reporting patterns (themes) within data” [23]. It has six steps: familiarization with the data, coding, generating initial themes, reviewing, defining and naming themes, and writing up the results. The themes identified in the analysis supply the meaning of each research question. Thus, our aim was to explore, in the themes, the patterns that the participants use to describe and understand what is happening. Finally, to evaluate the credibility of the results, we triangulated them in two ways, with the investigators and the data source triangulation [24]. In the first case, the two researchers supplied many observations that were compared and complemented, expanding the gathered information. In the second type of triangulation, we collected information from different people (care recipients, professionals, etc.), obtaining multiple perspectives and validation of the data.

## Results

### Description of the sample

**Fig. 2:**
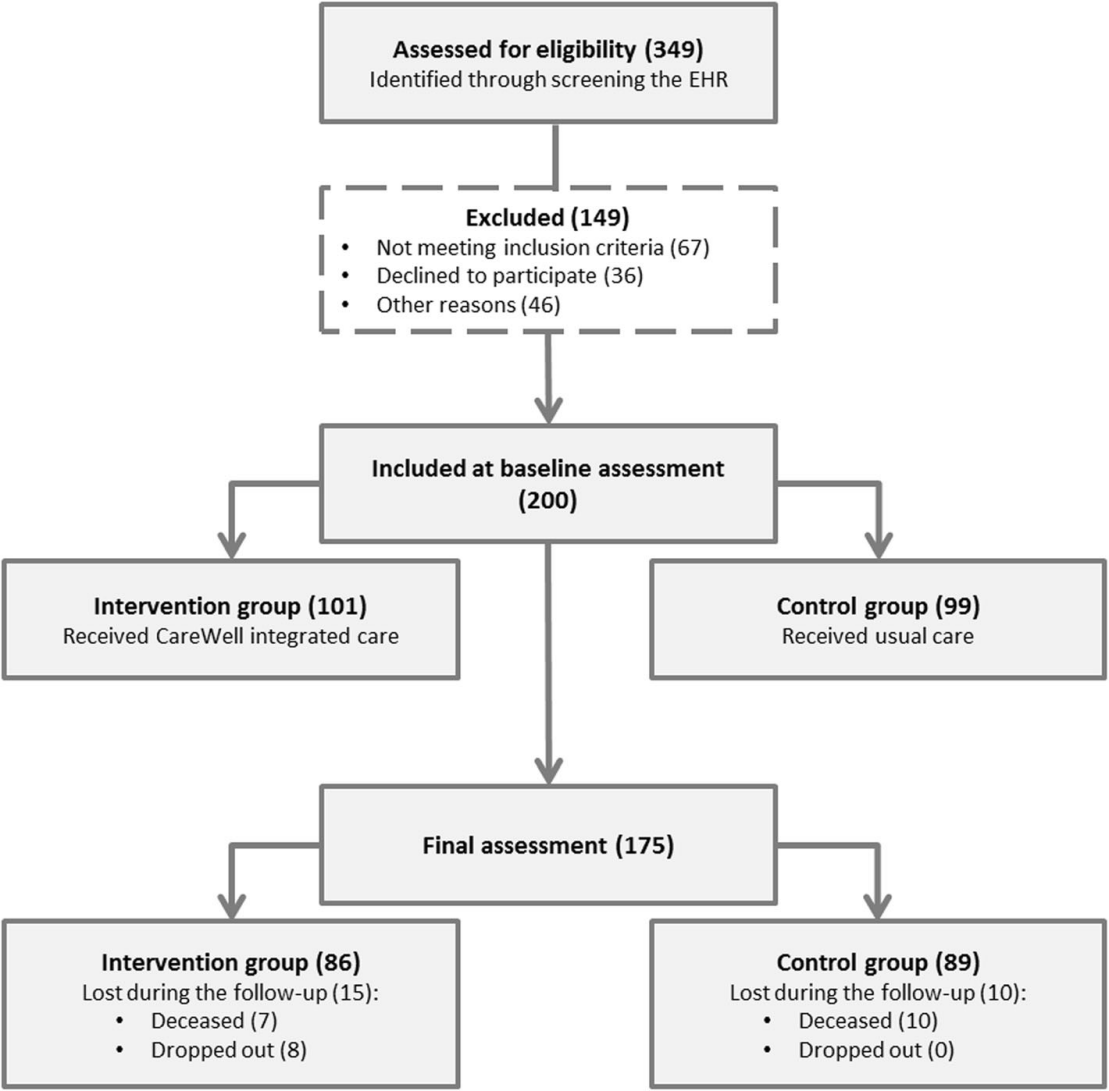
Diagram of the patients included. The flow of participants for control and intervention group, per site

Two hundred patients were recruited, 101 for the intervention group and 99 for the control. The flow of participants is shown in Fig. 2.

Baseline characteristics are shown in Table 1. There were no differences between sociodemographic and lifestyle characteristics in the intervention and control groups. The most prevalent diseases, apart from those in the inclusion criteria (COPD (88%), diabetes mellitus (85%) and CHF (79%)), were renal disease (46%) and peripheral vascular disease (32%).

The mean BMI of 30.4 indicated a high level of obesity, higher in the intervention group (*p*-value = 0.006) than in control. Regarding baseline mental health status, both groups presented mean values corresponding to normality, although close to depression levels (≥ 5 points), especially in the control group (*p*-value = 0.011).

**Table 1:** Baseline characteristics of the groups (intervention and control)

	Total	Intervention	Control	*p*-value
Sample size	200	101	99	
Age	79.4 (6.8)	79.6 (6.9)	79.2 (6.8)	0.716
Gender (female)	74 (37%)	34 (34%)	40 (40%)	0.401
Education level				0.094
Less than primary school	40 (20%)	16 (16%)	24 (24%)	
Primary school	118 (59%)	59 (59%)	59 (60%)	
Secondary school/Vocational training	33 (17%)	18 (18%)	15 (15%)	
University	8 (4%)	7 (7%)	1 (1%)	
Mobile use (Yes)	124 (62%)	58 (57%)	66 (67%)	0.230
Personal computer use (Yes)	20 (10%)	13 (13%)	7 (7%)	0.258
Smoking				0.336
Never	117 (58%)	58 (57%)	59 (60%)	
Former	67 (33%)	32 (32%)	35 (35%)	
Current smoker	13 (6%)	8 (8%)	5 (5%)	
Other	3 (1%)	3 (3%)	0 (0%)	
Body Mass Index	30.4 (5.5)	31.5 (5.6)	29.4 (5.2)	0.006
HbA1c	6.8 (1.2)	6.8 (1.3)	6.9 (1.1)	0.619
Creatinine	1.2 (0.5)	1.2 (0.6)	1.2 (0.5)	0.511
Barthel Index, median (Q1, Q3)	100 (80,100)	100 (80,100)	100 (80,100)	0.877
Geriatric Depression Scale	4.1 (3.1)	3.6 (2.7)	4.7 (3.3)	0.011
Age-adjusted CCI	9.7 (2.9)	9.6 (3.1)	9.9 (2.7)	0.478
Comorbidity				
Myocardial infarction	32 (16%)	19 (19%)	13 (13%)	0.351
Congestive heart failure	159 (79%)	82 (81%)	78 (78%)	0.700
Peripheral vascular disease	65 (32%)	31 (31%)	35 (35%)	0.617
Cerebrovascular disease	28 (14%)	11 (11%)	17 (17%)	0.295
Chronic pulmonary disease	176 (88%)	88 (87%)	88 (88%)	1.000
Mild liver disease	35 (17%)	13 (13%)	23 (23%)	0.091
Diabetes without complications	146 (73%)	66 (65%)	81 (81%)	0.019
Diabetes with complications	24 (12%)	12 (12%)	13 (13%)	0.979
Renal disease	92 (46%)	41 (41%)	52 (52%)	0.139
Any malignancy	24 (12%)	15 (15%)	9 (9%)	0.288
Moderate or severe liver disease	32 (16%)	18 (18%)	15 (15%)	0.727

Categorical data presented as frequencies and percentages (%) and continuous data as means and standard deviation, unless otherwise stated. CCI, Charlson Comorbidity Index; Comorbidity data show the incidence of comorbidity; (Q1, Q3), First and third quartile; HbA1c and creatinine only obtained for the patients reviewed to control specific diseases

### Change in the use of health services profile

**Fig. 3:**
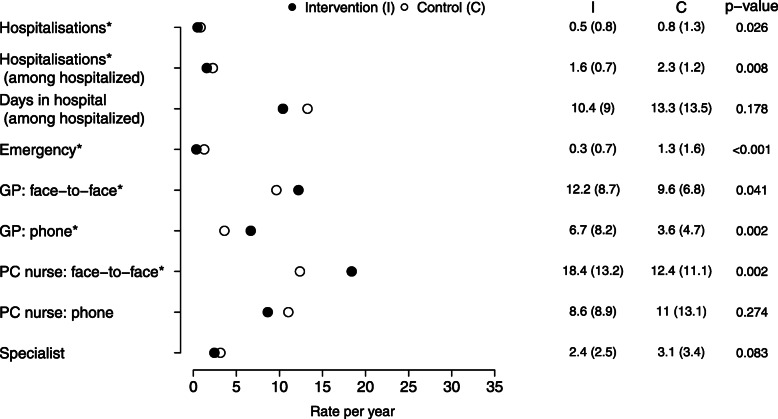
Use of health resources by each group, intervention and control. Data are presented as mean (standard deviation). The data represent the rate per year, considering the follow-up period for each patient. C, control; GP, general practitioners; I, intervention; PC, primary care. Differences between groups were measured using regression models. The models were adjusted by age, gender, baseline BMI value, and age-adjusted Charlson Comorbidity Index

After the deployment of the plan and during the follow-up period, statistically significant differences in the use of health resources were observed between the intervention and the control groups (Fig. 3). The rate of hospitalizations per year and the numbers of emergency visits were significantly higher in the control group than in the intervention group. The percentage of patients who were hospitalized at least once during the follow-up period was 31 and 37% in the intervention and control groups, respectively. Their hospital stay was longer for the control group; the mean number of days in the hospital was 13.3 (SD, 13.5), whereas the mean stay for the intervention group was 10.4 (SD, 9) days. This decrease in the use of hospital services was accompanied by an increase in the use of primary care services. The intervention group had more appointments with the GP, for both, face-to-face visits (*p*-value = 0.041) and phone contacts (*p*-value = 0.002). The number of face-to-face visits to the PC nurse was also higher in the intervention group, with statistically significant differences (*p*-value = 0.002).

### Clinical outcomes

Clinical effectiveness of the program was also observed to a certain extent (Table 2). BMI, blood glucose, blood pressure and oxygen saturation levels were significantly reduced in the intervention group. A decrease in the Barthel Index was observed both, in the intervention and the control group.

**Table 2:** Basal and final results and differences between the groups (intervention and control)

	Intervention	Control	β (95% CI)	*p*-value
Sample size	86	89		
BMI				0.047
Basal	31.4 (5.7)	29.2 (5.3)	–	
Final	30.6 (5.7)^a^	29.1 (5.4)	−0.5 (−1.1,-0.01)	
Heart rate				0.378
Basal	73.4 (11)	71.4 (11.4)	–	
Final	73.4 (12)	70.6 (12.2)	1.4 (− 1.8,4.6)	
Systolic blood pressure				0.046
Basal	132.3 (15)	138.1 (17)	–	
Final	127.5 (16)^a^	133.9 (16)^a^	−4.7 (−9.3,-0.1)	
Diastolic blood pressure				0.175
Basal	71.5 (9.4)	71.6 (10.1)	–	
Final	70 (9.6)	71.1 (11.1)	−1.9 (−4.8,0.9)	
Oxygen saturation				0.014
Basal	95.9 (2.1)	96 (2.1)	–	
Final	95.9 (2)	96.2 (2.3)	−0.9 (−1.6,-0.2)	
Blood glucose				0.049
Basal	120.5 (42)	127.7 (42)	–	
Final	112 (31)^a^	125.5 (47)	−12.6 (−25.2,-0.02)	
HbA1c				0.060
Basal	6.7 (1.2)	6.8 (1.1)	–	
Final	6.6 (1.1)	6.9 (1.3)	−0.33 (−0.7,0.01)	
Creatinine				0.309
Basal	1.2 (0.6)	1.2 (0.5)	–	
Final	1.3 (0.8)^a^	1.3 (0.6)^a^	0.05 (−0.1,0.04)	
Barthel index				0.561
Basal	89.5 (18.9)	88.2 (18.4)	–	
Final	86.9 (20.8)^a^	85.1 (20)^a^	1.0 (−2.5,4.6)	
Depression-GDS				0.656
Basal	3.3 (2.7)	4.7 (3.4)	–	
Final	3.2 (3)	4.3 (3.5)	−0.2 (−1.0,0.6)	

Data presented as mean, standard deviation or their corresponding 95% confidence interval (95% CI). ^a^ indicates pre–post differences within each group (intervention or control). BMI, body mass index; GDS, Geriatric Depression Scale, short form; β, beta coefficient of the intervention group, estimated from the regression model. Models were adjusted by baseline values of the specific outcome, baseline BMI value, follow-up period, age, gender and age-adjusted Charlson Comorbidity Index

### User perspectives

This section shows the results of the interviews for each of the main variables that were explored with patients, carers, and professionals. The main quotes from the stakeholders are included in Table 3.

**Table 3:** User perspective: quotes from the stakeholders

	STAKEHOLDERS			
	Patients	Carers	Professionals	
			Nurses	Clinicians
**Care plan**	*–* “As far as diabetes is concerned, this is a very old plan. Therefore, I have been going through a series of check-ups for many years.” *–*“At a certain point I promised my endocrinologist I would always co-operate whenever my help was required, in trials or anything, that I would be available… I’m here in Osakidetza to do what’s necessary.”	– “A person…who is a good patient (…). He/she would let us do anything.” – “She is aware and helps us as much as she can.”	– “In particular, follow-up, control of acute exacerbations as well as monitoring several chronic illnesses; and anything that may come up in acute illnesses”. “And also, above all, I try to educate them in health issues related to chronic diseases” – “What we do is care for patients with a fragility level of 4 to 5 because we believe they can benefit the most from this specific home care.”	– “Normally care on demand, vaccination campaigns… The nursing department does nursing check-ups and then provides home care assistance.”
**Impact**	*The question was only addressed to the professionals*	*The question was only addressed to the professionals*	– “The first part of the study included very specific health education lessons which had to be delivered every week, picking up the thread, and that was the hardest.” – “It had a positive impact on my work and the rest of the team’s work.” – “…we have got used to working in a way, which, in my view, is correct, improving the prevention and promotion instead of only providing care at the critical times...”	– “When we screened the list of patients that they had sent us, we had to review it, and it increased the workload” – “Well, I actually collaborated in patient selection and in the follow-up of any decompensation or problems; but the person in charge of their education was the nurse, during the check-ups.” – “What were the differences in comparison to the path we had used before? Well, I believe two main things had been lacking: one is nursing and the other proactivity.”
**Changes**	– “Since the last time, when I had a build-up of fluid approximately last February or March, I have changed my diet since then and adapted it to what I do now, and all those things.” – “Maybe more assistance… I recorded everything I did every month and handed it to them, and that was certainly another follow-up” – “As you know more things, you see things you didn’t notice before.”	– “The nurse calls me when she finds it convenient or when she/he looks at the report or whatever, and she/he usually calls me”. – “Attention at the health center was..., there haven’t actually been any changes, (…) excellent from the start.”	– “We still have a lot to do…we are learning to coordinate, working on it; however, we all still have to remember that there is someone else on the other side who works like me in another field and needs to know what I think and what I’m going to do.” – “They are used to us looking after them and making decisions …we can try to teach them the warning signs…rather than basic daily control, that none of them have had before.” – “What I noticed is that the health professionals used to act in acute situations, without prevention or promotion.”	– “Over the last year or two, we have developed a much closer contact with specialist care and, in particular, with internal medicine.” – “I believe that they are more responsible, yes, they know the warning symptoms and do not wait to start feeling fatigued before coming to see us.”
**Care coordination**	– “Let me put it like this, since its implementation, since everyone I deal with from Osakidetza can access the central PC data, it has improved”.	– “At least you have coordination (…), you are not helpless.”	– “…I have noticed that the healthcare professionals used to react to an acute process without prevention or promotion and this program, (...) this way of working…has helped them to move towards the first phase.”	– “Over the last year or two, we have had a much closer relationship with specialist care and, in particular, with internal medicine.”
**Expectations- Satisfaction**	– “I felt confidence and an unknown quantity which was, will it work or not? And was confident I would do it, just in case it works.”	– “No, our expectations were not for him to improve but to help others improve.” *–* “…Better supported, you think, well, I can call and ask because there are people there.”	– “That’s what I wanted, to see the benefits of follow-up and the prevention, promotion, patient’s empowerment at the primary care level, so they can experience it the way I do.”	– “And if the study were going to be more important and more international, it would add more validity to the one we have had”. – “Another expectation: will this really improve the patient quality of life and avoid admissions?”
**Deployment of the program**	– “As soon as people become aware of the severity of their situation, have a little follow-up and see they are being listened to, it’s highly positive.”	– “I believe we should all be involved to improve the entire generation, not just ourselves.”	– “The problem for the professionals is that we don’t know what each of us does.” – “Increase the patient’s empowerment, not when the illness is so advanced that despite all the empowerment that we want to give them, they already need all resources available, but earlier.” – “More home visits, more patient education, more listening to the person to identify his/her needs and using new technologies; these are all required.”	– “For a professional, everything new generates an expectation, let’s see what happens...what this is going to entail over time, see what this means to us because our main problem is time”. – “I don’t believe we need more resources for this, I think we have to get organized first, which is what we’re trying to do.”
**Use of the ICT**	– “If I want to, I can have it via the Internet (…) Yes, but I actually don’t want to, if I have my doctors working on that.”	– “I’m not exactly an IT expert, but for those things, I keep a piece of paper and take it everywhere; then I do my own translation.”	– “The problem is we’re dealing with very elderly patients. As that’s a handicap, we need to find support among the carers.” – “That’s why, for me, starting these programs with young people is essential because in the long-term, by the time patients are 60, they will have already covered all that journey.”	– “If my patients have access to contact through the health files and each time they write to me, they will book an appointment in my diary and have time allocated to them; I could do it perfectly, and that would greatly help everyone.” – “For professionals, it promotes sharing knowledge, cooperation, networking.”

Care plan: Patients and carers seemed to be firmly involved in their health care plans, which was partly due to the close relationship that they developed with their primary care nurses and doctors. The largest workload was carried by the nurses, as they controlled and revised the main parameters that the patients had to monitor, and they were also responsible for the patient education.

Impact: Nurses, clinicians, and managers were asked about the impact of the integrated care program on their daily work with patients. For clinicians, the hardest task to perform was at the beginning of the study, when they had to revise the list of patients to select the study candidates. They all agreed that the nurses did the hardest work from the outset of the study. The nurses had to conduct the weekly health education sessions and during the progress of the study they had to call and visit patients more frequently than before the start of the program. The main changes triggered by the implementation of the program were related to nursing roles, proactivity, and patient empowerment.

Changes: Nurses were perceived as more alert and watchful, more closely following-up the health status of the patients. The program increased their awareness of the illnesses and the signs they must control to avoid deterioration. The opinion of the professionals was that they had already been well coordinated at the primary care and other levels. Their perception of the patients was that the participants changed their attitude and became more responsible and autonomous.

Care coordination: Patients and carers perceived the health professionals as well-coordinated, and thought that, since the integration of the electronic medical record, the management of the information has improved. In most cases, the between-level coordination had been initiated before the implementation of this integrated program as the Basque Health Service had been working with pluripathologic patients in other projects.

Expectations–satisfaction: Patients and carers did not have too many expectations apart from those related to better control of their diseases and the health care regimen they had to follow. For the nurses and clinicians, this project meant a continuation of the work in which they had been involved already. Globally, the participants were satisfied with the experience: the patients and carers because they felt more controlled, more secure; and clinicians and nurses because they could confirm/validate what they were doing.

Deployment of the program: The professionals were asked about the main barriers and the requirements for this implementation. Some of them thought that the program has changed the way they work and increased their workload. They did not know enough about the work of other professionals, which sometimes made them reluctant to share their own knowledge and experiences. For a general deployment of the program, an increase in human resources and time available for the implementation was considered necessary.

Use of ICT: The professionals believed that ICTs could help in coordination and collaboration with others. However, they thought that this technology could not be used with the patients participating in this project as they were quite old and lacked technical experience. The Personal Health Folder could be a very good tool to help in solving this problem. Still, it is underused because of lack of awareness and because some of its functionalities are not completely implemented.

## Discussion

There is little high-quality evidence available on the deployment of integrated care models [25]. The CareWell project answers the need to assess this kind of intervention. It provides the evaluation of the implementation and the impact of a new integrated care program in a large sample of older patients with multimorbidity. The program follows a complete integrated care model that emphasizes care coordination and patient empowerment.

The CareWell process furthered the reform of the Basque Health System, which had been started some years before [5]. It included several key elements referred to in the integrated care literature [26] but not always easy to incorporate into clinical practice [27, 28]. CareWell intervention contained a large range of new elements, such as the patient stratification, the establishment of new roles (e.g., the care manager and the reference internist), and the multidisciplinary working team sharing explicit decision making information, such as, assessment tools and scales, communication protocols and care plans, and patient and carer training and skill development, among others.

The study shows different utilization of health resources in the intervention and control groups. Patients in the intervention group had fewer hospital admissions and made fewer emergency visits, whereas they had more contacts with their GPs, especially by phone, and more face-to-face visits with their PC nurse. This was consistent with other studies [29–31]. This change in the use of services was also captured using qualitative techniques and noted by professionals and patients themselves. The shift in the use of health resources from the hospital to the primary care has also been observed for the overall CareWell project [32]. This change in the workload allocation, displaced towards primary and home care, can be seen as an indirect confirmation of effectiveness and quality improvement [33]. The proposed care pathway pivoted on a new prominence and role of primary care nurses [34], designed to improve between-level coordination. This strengthened role resulted in an increase in face-to-face PC nurse visits. The number of phone contacts with the PC nurse did not change. This might be a result of the new process to improve continuity of care of discharged hospital patients, which had also been deployed in the control organizations.

The ICT tools may have a positive effect on the continuity of care, and communication between health providers. Although both the intervention and control groups benefited from the well-developed ICT tools such as EHR and e-Prescription, the ICT on its own is not sufficient. Health ICT-enabled coordination with a multidimensional approach is needed [10, 12]. Organizing care involves marshaling the personnel and other resources to carry out all the required patient care activities; this is often managed by sharing the information among the individuals responsible for different aspects of care.

As in other studies [35, 36], most of the stakeholders expressed a high degree of satisfaction with this integrated care model. However, one important barrier was identified: PC professionals felt that the program, as has been defined increased their workload. This rise in the primary care workload and leadership requirements might not be sustainable using solely the existing resources [37]. An effort to better fund Primary Care has been observed in the last couple of years, improving staff population rates.

Our study showed scarce effect on some health-related outcomes such as health-related quality of life in terms of mental functioning, medication use, functional status, mortality or physical functioning [35]. However, the CareWell intervention improved some clinical outcomes, such as BMI, blood glucose, blood pressure, and oxygen saturation levels. The decrease in the value of the Barthel Index, observed in both groups, is probably attributable to the passage of time, as the functional independence necessary for daily living activities is expected to decrease in such complex aged patients.

The improvements in some clinical aspects in combination with the change in the use of health resources could be due also to the patient empowerment pathway implemented in the intervention group. Patient empowerment should improve the way patients take care of themselves, help them to interact with health care services, and gain ownership of their health [38]. The qualitative results of the intervention suggest that the patients felt more secure and empowered in the management of their health and were more satisfied.

The results of the implementation of the CareWell Program in the Basque Country are consistent with previous reports suggesting that initiatives to improve the care of people with long-term conditions enhance their satisfaction with care, quality of life, and in some cases, use of health services [39, 40]. Above all, the implementation of the CareWell project has improved the integration in the Basque health system. Around 6200 patients benefited from the proposed integrated care pathway; it has been adapted and scaled up by the ACT@SCALE project [41]. Furthermore, the comprehensive patient empowerment program KronikON, developed specifically for CareWell, is now available to patients and their informal carers through the Electronic Health Folder and the Osakidetza web portal [18].

However, there is a shortage of standardized, validated tools for routine use in the evaluation of integration outcomes. This makes the measurements and comparisons of the effects of integration at the system, provider, and patient levels remain challenging [42]. In most cases, a mixed methodology approach is required [43]. Then, the results obtained by quantitative and qualitative analyses can be combined, adding breadth and perspective to the process and helping to understand the outcome.

Some limitations of the study should be discussed here. The main limitation was the lack of random allocation, as the subjects willing to participate may have not been representative of the target population; nevertheless, the two groups were comparable at baseline regarding the main results variables. The relative importance of different components and roles in integrated care was not been examined in depth. One could argue, however, that the combined effects, rather than individual elements, are the decisive factors leading to the success of integrated systems [10].

The number of interviews could be considered as not sufficient to be representative. However, the goal of qualitative research is the development of concepts that help to understand social phenomena in their natural (rather than experimental) settings, giving due emphasis to the meanings, experiences, and views of all the participants [44]. The purpose of this study was to focus on the implementation constructs of real-life contextual understanding, multi-level perspectives, and cultural influences so data were collected from key informants in the main stakeholder groups, who served as expert sources of information [45, 46].

## Conclusions

The implementation of the CareWell integrated care model changed the profile of health resource utilization, strengthening the key role of primary care in the management of older patients with complex multimorbidity. Moreover, the program achieved a reduction in the number of emergency visits and hospitalizations.

The satisfaction with this model of care was high among all the stakeholders, patients, carers, and health professionals. This model of care should be considered when targeting this kind of complex populations.

## Supplementary information

**Additional file 1.** Qualitative interview templates. Templates of the qualitative semi-structured interviews for each stakeholder.

## Data Availability

The datasets used and analyzed during the current study are available from the corresponding author on reasonable request.

## Abbreviations

BMI: Body mass index
CHF: Chronic heart failure
COPD: Chronic obstructive pulmonary disease
EHR: Electronic Health Records
GDS: Geriatric Depression Scale
GP: General Practitioner
ICO: Integrated care organizations
ICT: Information and communication technologies
NHS: National Health Services
Osakidetza: Basque healthcare system
PC: Primary care
Q1: First quartile
Q3: Third quartile
SD: Standard deviations
