# Supplementary material for: Impact of the CareWell integrated care model for older patients with multimorbidity: a quasi-experimental controlled study in the Basque Country
Source: BMC Health Serv Res. 2020 Jul 3;20:613. doi: 10.1186/s12913-020-05473-2 (PMC7333301; doi:10.1186/s12913-020-05473-2)
Supplement: Supplementary file 1 — Additional file 1. Qualitative interview templates. Templates of the qualitative semi-structured interviews for each stakeholder. [file 12913_2020_5473_MOESM1_ESM.docx]

**Patients’ interview**

Presentation

1. Could you please shortly present yourself: your name and your place of residence?

Care plan

2. Do you have an overall care plan for the management of your disease? Do you feel involved in your disease care plan? Can you tell me more about your role/participation in this care plan?

Coordination of your care

3. Thinking about all the health services you have used in the last months, how do you experience or think your care has been coordinated (For example, the way different doctors, nurses, social workers and organizations work together)?

4. Which changes have you noticed in the overall care you have been receiving lately? (Since….months, when you entered in the CareWell program).

5. Have you perceived any attitude change in the professionals since the start of this new program? If yes, which attitudes have been modified?

6. Please, describe how the new integrated care has generated a change in your attitude in relation to your illness. For instance: Has it helped you understand better your illness? How? / Does it help you cope better with your illness? How? / Does it make you more confident about your health?

7. Which were your expectations with the integrated care plan when asked you to participate using it? Are you satisfied with it?

8. Do you think that this new integrated care plan should be offered to all the patients in your situation? Why?

Management of your disease by the use of ICT

9. Please, explain how the technology (mobile phone, tablet, others…) is being used for supporting your disease management. Have you found any difficulties? Could you tell me about your experience with these technology resources?

10. Do you and/or your family members/caregivers have access to your health data? If so, what do you use the information for? If not, would you like to do so?

11. Do you feel safe/cared using this technology? Have you experienced any inconvenience (annoyance/adverse events/unsafety) using the application *(changed by the word that best suits in each site)*? If yes, could you explain these inconveniences?

**Caregivers’ interview**

Presentation

1. Could you please shortly present yourself: your name, your place of residence and the person you are caring for?

Care plan

2. Does he/she have an overall care plan for the management of his/her disease? Do you think he/she feels involved in his/her disease care plan? Can you tell me more about his/her role/participation in this care plan?

Coordination of care

3. Thinking about all the health services he/she has used in the last months, how do you experience or think the care has been coordinated (For example, the way different doctors, nurses, social workers and organizations work together)?

4. Which changes have you noticed in the overall care he/she has been receiving lately? (Since….months, when he/she entered in the CareWell program).

5. Have you perceived any attitude change in the professionals since the start of this new program? If yes, which attitudes have been modified?

6. Please, describe how the new integrated care has generated a change in his/her attitude in relation to his/her illness. For instance: Has it helped him/her understand better the illness? How? / Does it help him/her cope better with the illness? How? / Does it make him/her more confident about his/her health?

7. Which were his/her expectations with the integrated care plan when asked him/her to participate using it? Do you think he/she is satisfied with it?

8. Do you think that this new integrated care plan should be offered to all the patients in his/her situation? Why?

Management of the disease by the use of ICT

9. Please, explain how the technology (mobile phone, tablet, others…) is being used for supporting his/her disease management. Have you found any difficulties? Could you tell me about your experience with these technology resources?

10. Does he/she and/or his/her family members/caregivers have access to his/her health data? If so, what does he/she use the information for? If not, would he/she like to do so?

11. Does he/she feel safe/cared using this technology? Has he/she experienced any inconvenience (annoyance/adverse events/unsafety) using the application *(changed by the word that best suits in each site)*? If yes, could you explain these inconveniences?

**Professionals’ interview**

Presentation

1. Please introduce shorty yourself (name and working position)? How long have you worked in your current job?

2. Can you describe the care you provide to frail multimorbid patients?

Impact of the Integrated Care Program

3. Which has been the impact of the Integrated Care Program on the daily work with the patients, on your routine, on your workflow?

4. How the implementation of the Integrated Care Program has changed your relation with other professionals? How has it improved your ability to coordinate with other professionals? (Within the hospital / Between hospital and GP´s and nurses? / Others (social care…).

5. How the Integrated Care Program has changed your relationship with the patient? Please describe.

6. How has the Integrated Care Program changed the attitude of the patient towards his/her illness and the way he/she manages it? For instance, do you perceived patients more responsible/autonomous about their health? If yes, in which way?

7. Which do you perceive that have been the main resistances/barriers to the new care model?

8. Which were your expectations with the application before starting? Are you satisfied with it?

9. What do you think is needed to spread this model to the whole organization? Are new resources necessary? If yes, which kind of resources?

Evaluation of the ICT

10. In your point of view, what are the benefits or downsides using ICT to access to patient information? And which are the benefits or downsides using the ICT to communicate with the patient?

11. Do you feel comfortable using this technology? Have you experienced any inconvenience using the application *(changed by the word that best suits in each site)*? If yes, could you explain these inconveniences?

**Managers’ interview**

Presentation

1. Please introduce shorty yourself (name and working position)? How long have you worked in your current position?

2. Can you describe the care provided to frail multimorbid patients in your organization?

Impact of the use of Integrated Care Program

3. Has the Integrated Care Program had any impact on the daily work with the patients, on the routine of the professionals, on their workflow and attitudes? If yes – will you please describe this impact?

4. How the implementation of the Integrated Care Program has changed the relation between care levels? (Within the hospital / Between hospital and GP´s and nurses? / Others (social care…)

5. How the implementation of the Integrated Care Program has changed the services use pattern (less emergency visits, home care nurse visits …)?

6. How would you describe the collaboration in implementing the Integrated Care Program in your organization?

7. Which do you perceive that have been the main resistances/barriers to the new care model?

8. What do you think is needed to spread this model to the whole organization? Are new resources necessary? If yes, which kind of resources?

Evaluation of the ICT

9. In your point of view, what are the benefits or downsides using ICT to coordinate, plan, inform and communicate about the patient?
